# R2R3-MYBs in Durum Wheat: Genome-Wide Identification, Poaceae-Specific Clusters, Expression, and Regulatory Dynamics Under Abiotic Stresses

**DOI:** 10.3389/fpls.2022.896945

**Published:** 2022-06-20

**Authors:** Emanuela Blanco, Pasquale Luca Curci, Andrea Manconi, Adele Sarli, Diana Lucia Zuluaga, Gabriella Sonnante

**Affiliations:** ^1^Institute of Biosciences and Bioresources, National Research Council (CNR), Bari, Italy; ^2^Institute of Biomedical Technologies, National Research Council (CNR), Milan, Italy

**Keywords:** R2R3-MYB transcription factors, durum wheat, abiotic stress, Poaceae-specific clades, gene regulatory network

## Abstract

MYB transcription factors (TFs) represent one of the biggest TF families in plants, being involved in various specific plant processes, such as responses to biotic and abiotic stresses. The implication of MYB TFs in the tolerance mechanisms to abiotic stress is particularly interesting for crop breeding, since environmental conditions can negatively affect growth and productivity. Wheat is a worldwide-cultivated cereal, and is a major source of plant-based proteins in human food. In particular, durum wheat plays an important role in global food security improvement, since its adaptation to hot and dry conditions constitutes the base for the success of wheat breeding programs in future. In the present study, a genome-wide identification of R2R3-MYB TFs in durum wheat was performed. MYB profile search and phylogenetic analyses based on homology with Arabidopsis and rice MYB TFs led to the identification of 233 R2R3-TdMYB (*Triticum durum* MYB). Three Poaceae-specific MYB clusters were detected, one of which had never been described before. The expression of eight selected genes under different abiotic stress conditions, revealed that most of them responded especially to salt and drought stress. Finally, gene regulatory network analyses led to the identification of 41 gene targets for three TdR2R3-MYBs that represent novel candidates for functional analyses. This study provides a detailed description of durum wheat R2R3-MYB genes and contributes to a deeper understanding of the molecular response of durum wheat to unfavorable climate conditions.

## Introduction

Transcription factors (TFs) are required for the transcriptional control of gene expression. Thanks to this regulation and to other regulatory mechanisms, tissue specific-gene expression and response to particular cell signaling pathways are guaranteed. Transcription factors are characterized by the presence of a DNA binding domain (DBD) that allows positive or negative regulation of their target genes. Based on the characteristics of the DBD, TFs are classified in different families (Latchman, 1997).

In plants, the MYB TFs represent one of the biggest TF families (Martin and Paz-Ares, 1997; Riechmann et al., 2000; Stracke et al., 2001). The MYB highly conserved DBD consists of 1–4 tandem repeated imperfect amino acid (aa) sequences (R), each of approximately 50–53 aa forming three α-helices. The second and third helices adopt a helix-turn-helix structure with a hydrophobic core formed by three regularly distributed tryptophan (or phenylalanine) residues; the third helix directly intercalates the major groove of the DNA (Ogata et al., 1992, 1996; Dubos et al., 2010; Zhang et al., 2012a). Based on the repeat number, four subfamilies have been identified: R1-MYB or MYB-related with a single or a partial MYB repeat, R2R3-MYB with two repeats, R1R2R3-MYB with three repeats and 4R-MYB with four R1/R2-like repeats (Dubos et al., 2010; Katiyar et al., 2012). More than 80 *R2R3-MYB* genes were initially identified in the *Arabidopsis thaliana* genome, representing the major contribution to the large size of the MYB family (Romero et al., 1998). Over the years, thanks to a large number of studies and to the advancement of new “omics” technologies, these data have been continuously updated in Arabidopsis and confirmed in other plant species, including grapevine (*Vitis vinifera*), petunia (*Petunia hybrida*), apple (*Malus domestica*), maize (*Zea mays*), rice (*Oryza sativa*), bread wheat (*Triticum aestivum*) (Dubos et al., 2010; Du et al., 2015; Jiang and Rao, 2020; Wei et al., 2020; Xiao et al., 2020). The multiplicity of *R2R3-MYB* genes is related to the functional diversity of these genes in plants (Bolser et al., 2017). Indeed, their involvement in various specific plant processes, such as secondary metabolism, cell cycle, development, and responses to biotic and abiotic stresses, has now been confirmed in several studies (Stracke et al., 2001; Dubos et al., 2010; Blanco et al., 2018; Millard et al., 2019).

The implication of MYB TFs in the tolerance mechanisms to abiotic stress is particularly interesting for crop breeding, since adverse environmental conditions, such as drought, high salinity, high or low temperature, absence or excess of nutrients in general, can negatively affect growth and productivity (Mittler, 2006; Zhang et al., 2012a). Thus, in recent years, researchers are increasingly focusing their studies on the identification of MYB TFs involved in stress tolerance in Arabidopsis and in crop species (An et al., 2018; Zhao et al., 2019, 2020b; Deng et al., 2020; Fang et al., 2020; Wang et al., 2020; Zhang et al., 2020). In cereals, several studies show the implication of MYB TFs in the abiotic stress response. For instance, a wheat TaMYB30-B improves drought stress tolerance in transgenic Arabidopsis through the regulation of stress-responsive genes (Zhang et al., 2012b). Overexpression of rice *OsMYB55* resulted in improved plant growth under high temperature through the regulation of various genes involved in amino acids metabolism, including glutamine synthetase (OsGS1;2) glutamine amidotransferase (GAT1) and glutamate decarboxylase 3 (GAD3) (El-kereamy et al., 2012). The wheat *TaODORANT1* overexpression upregulated the expression of several stress-related genes in response to both drought and salt stresses in transgenic tobacco (Wei et al., 2017). Wheat *TaMpc1-D4* overexpression reduced drought tolerance in transgenic Arabidopsis and its silencing increased drought tolerance (Li et al., 2020c). The overexpression of *TaMYB344* in transgenic tobacco enhanced drought, heat, and salt stress tolerance (Wei et al., 2020). Moreover, several MYB genes were found to be regulated by nitrogen deprivation in wheat (Curci et al., 2017; Wang et al., 2019).

Durum wheat [*Triticum turgidum* subsp. *durum* (Desf.) Husn.] is an important cereal species, mainly cultivated in the Mediterranean countries, characterized by scarcity of rain, with critical problems such as drought, salinity and low amount of inorganic matter (Xynias et al., 2020). The publication of the fully assembled tetraploid genome (BBAA) of the durum wheat cultivar Svevo (Maccaferri et al., 2019) provides the opportunity of studying gene families at the genome scale.

The objective of this study was to identify R2R3-MYB transcription factors possibly involved in the regulation of abiotic stress response in durum wheat. To this aim, we performed a genome-wide identification of the R2R3-MYB gene family in durum wheat and focused on eight selected genes by analyzing their expression under different stress conditions, including salinity, drought and nitrogen (N) deprivation. Furthermore, we explored their regulatory dynamics to identify gene targets that could serve to accelerate plant breeding.

## Materials and Methods

### Identification and Phylogenetic Analysis of Durum Wheat R2R3-MYBs

To identify R2R3-MYB TFs in durum wheat, Arabidopsis and rice MYB protein sequences were downloaded from Plant TF Database^1^ (PTFD; Jin et al., 2017) and used as queries for BLASTP searches against the durum wheat cv. Svevo genome (available at https://wheat.pw.usda.gov/jb/?data=/ggds/whe-svevo2018), for *A. thaliana* (e-value 1E-90, 80% coverage) and *O. sativa subsp. japonica* (e-value 1E-50, 60% coverage), separately.

The Hidden Markov Model (HMM) profile for MYB DNA-binding domain PF00249^2^ was used as query to run HMMER^3^ analyses (HMMER 3.1b2, cutoff e-value: 0.01) against (1) the output of previous BLAST, and (2) all MYB annotated proteins.^4^ The final HMMER output was subsequently checked for the presence and the integrity of the characterized MYB DBDs using the National Center for Biotechnology Information (NCBI) Conserved Domain Database (CDD) (Lu et al., 2020).

To select only putative R2R3-MYB TFs, a multiple sequence alignment (MSA) was performed by MAFFT online service^5^ (version 7; Katoh et al., 2019) using MAFFT alignment with L-INS-i preset. The selected full-length amino acid sequences were aligned with Arabidopsis MYB TFs retrieved from Plant TFDB and annotated according to Katiyar et al. (2012) and Jiang and Rao (2020). Manual inspection was carried out using Jalview (Waterhouse et al., 2009) to confirm the presence of both R2 and R3 repeats, and sequences with incomplete MYB repeats and/or lacking more than two highly conserved tryptophan residues were discarded (Yanhui et al., 2006; Du et al., 2015). Moreover, durum wheat TRITD3Av1G223560 with ambiguous bases (Ns) in its CDS sequence and very low expression (<1 TPM) was removed from the analyses.

R2R3 domains of putative durum wheat R2R3-MYBs were extracted and aligned with MAFFT (L-INS-i) to generate sequence logos. R2R3 start and end amino acid positions were annotated and related nucleotide positions were located on both coding and genomic sequences.

Phylogenetic studies based on maximum likelihood (ML) analyses were performed using W-IQ-TREE (Trifinopoulos et al., 2016), with 1,000 ultrafast bootstrap replicates. Phylogenetic trees were imported and annotated with iTOL v6 (Letunic and Bork, 2019).

Members of Poaceae-specific and Poaceae-unique clades (Jiang and Rao, 2020) were identified through search on PLAZA 4.5 database for orthologous gene family identification (Van Bel et al., 2018). Motif search, comparison and enrichment were performed with *universalmotif* (Tremblay, 2019) and MEME suite (Bailey et al., 2015). Identified members were finally filtered by manual inspection of the integrity of R2R3 domains.

### WebLogo and MEME Analyses

A graphical representation for the amino acid distribution of durum wheat MYB R2 and R3 repeats was generated by submitting the MAFFT L-INS-i multiple sequence alignment of R2R3 domains to WebLogo with default parameters (Crooks et al., 2004).

MEME (Bailey et al., 2015) was used to identify durum wheat R2R3-MYB conserved auxiliary motifs, by setting the maximum number of motifs to 36 with a minimum width of six and a maximum width of 53 amino acids, while the other parameters were set to default values.

### Chromosomal Location, Gene Structure, Protein Properties, and Cellular Localization

The length of each chromosome and the chromosomal location of the identified R2R3-MYB genes were obtained from Ensembl Plants (Yates et al., 2022), using biomaRt (Durinck et al., 2009). Based on this information, chromoMap (v0.2) (Anand and Rodriguez Lopez, 2022) was used to create a chromosome physical map, on which the identified genes were annotated. The newly identified durum wheat R2R3-MYB TFs were named according to their chromosomal location from genome A to U (unannotated) and from chromosome 1–7.

The exon/intron structures were visualized with Gene Structure Display Server (GSDS) (Hu et al., 2015), using the coding sequences (CDS) and corresponding genomic sequences retrieved from Ensembl Plants. The retrieved genomic R2R3 start and end nucleotide positions were also submitted to GSDS.

Protein sequences were used to predict the isoelectric point (pI) and molecular weight (Mw) of each durum wheat R2R3-MYB using the ExPASy-Compute pI/Mw tool (Artimo et al., 2012). The subcellular localization of each protein was predicted using the CELLO v2.5 Server (Yu et al., 2004, 2006).

### Plant Material and Growth Conditions

The durum wheat cv. Svevo (CIMMYT Selection/Zenit, released in 1996, code number 4417 of the Italian Catalog, Ministry of Agriculture and Forestry-MIPAAF) was used in this study. Seeds were embedded in petri dishes with wet filter paper and kept at 4°C for germination. After 10 days, seedlings were moved to a growth chamber under 8 h light at 13°C and 16 h dark at 11°C, with a relative humidity (RH) of 50% for 3 days. Once the epicotyls were 0.5 cm, seedlings were transferred into net pots containing 80% agriperlite and 20% expanded clay and grown in a hydroponic system. A standard nutrient solution was prepared starting from tap water as in Curci et al. (2017) and containing 0.1 mM KH_2_PO_4_, 0.65 mM MgSO_4_⋅7H_2_O, 2 mM Ca(NO_3_)_2_⋅4H_2_O, 0.75 mM K_2_SO_4_, 10 mM H_3_BO_3_, 1 mM MnSO_4_, 1 mM ZnSO_4_, 5 mM CuSO_4_, 0.05 mM [(NH_4_)_6_Mo_7_]_2_⋅4H_2_O, and 100 mM Fe-EDTA. The root system was kept submersed in the nutrient solution, which was continuously aerated with an air pump, maintained at pH 5.7 with 0.1 N H_2_SO_4_ and refreshed every 2 days. For salt stress assay, 72 Svevo plants (3 plants per pot, for each biological replicate) were grown for about 2 weeks in the above conditions, until the Z14 growing stage was reached (Zadoks et al., 1974). At this developmental stage, the plants were divided into two groups: 36 plants were transferred to a 250 mM NaCl solution, in order to induce salinity stress (NaCl-stressed) and the other 36 plants were maintained in the standard nutrient solution as control plants (NaCl-control). For drought stress experiment, the same procedure was followed to grow 72 plants until they reached the Z14 stage. Subsequently, 36 plants were transferred to a 16.1% PEG 6000 solution (PEG-stressed) containing 10% macronutrient solution, 0.1% of micronutrient solution, 161 g/L of PEG 6,000 and 0.6 ml of sulfuric acid to reach the desired pH. The other 36 plants remained in the standard nutrient solution (PEG-control). To perform the nitrogen starvation experiment, plants were initially grown as above in standard N conditions (Zuluaga et al., 2018). At the Z14 stage, half of the plants were transferred into a N-deprived solution, where Ca(NO_3_)_2_⋅4H_2_O was replaced with 1.77 mM of CaCl_2_⋅2H_2_O (N-stressed), in order to impose a short term N stress as described in Zuluaga et al. (2018). Roots and leaves were collected from control and stressed plants at 2, 6, 12, and 24 h after salt or drought stress treatment and at 6, 12, 24, and 48 h after nitrogen stress experiment, immediately frozen in liquid nitrogen and stored at −80°C before use.

### Quantitative Real-Time PCR

Total RNA was extracted from roots and leaves of stressed (NaCl, PEG or N) and corresponding control seedlings, using a NucleoSpin RNA Plant kit (Macherey-Nagel, Düren, Germany) and treated with rDNAse (Macherey-Nagel, Germany) according to the manufacturer’s specifications. RNA concentration was determined using NanoDrop ND-1000 spectrophotometer (Thermo Fisher Scientific, Wilmington, DE, United States). For cDNA synthesis, 1 μg of total RNA was reverse transcribed using a PrimeScript RT reagent kit with gDNA eraser (Takara Bio, Inc., Otsu, Japan). Specific primer pairs were designed with Primer3Plus program^6^ (Supplementary Table 1). Standard Sanger sequencing of the obtained amplicons was performed to validate primer specificity. For quantitative real-time PCR (qPCR), the total volume for each reaction was 10 μl, containing 5 μl PowerUp SYBR Green Master Mix (Thermo Fisher Scientific, Vilnius, Lithuania), 0.5 μl cDNA and 200 nmol for each primer, adjusting the volume with distilled water. The gene coding for RNase L inhibitor protein [RLI(a)], previously tested for stability in our experimental material, was used as an internal reference (Curci et al., 2017, 2018; Zuluaga et al., 2017, 2018). Gene quantification was performed using a StepOnePlus Real-Time PCR Systems (Applied Biosystems, Foster City, CA, United States). The thermal cycling conditions were set as follows: 95°C for 3 min, followed by 40 cycles of 95°C for 15 s and 60°C 30 s, with data collection at 60°C. Melting curve analysis was used to verify the specificity of qPCR amplification. Three biological replicates, each with three technical replicates, were analyzed and each qPCR experiment was repeated twice to ascertain reliability of the results. Linear data were normalized to the mean Ct of the RLI(a) gene and the relative expression ratio was calculated using the 2^–ΔΔCt^ method (Livak and Schmittgen, 2001) at each time point for each stressed sample with respect to the corresponding control (non-stressed sample). Standard deviation was calculated, and Student’s *t*-test for statistical significance was performed.

### TdR2R3-MYB Gene Regulatory Networks Under Abiotic Stress Conditions

A deep screening of the scientific literature was performed to retrieve durum wheat RNA-seq experiments on abiotic stresses and with raw data available on Sequence Read Archive (SRA) database. Obtained raw RNA-Seq data were processed with Trimmomatic (v0.38) (Bolger et al., 2014) to perform adapter clipping and quality trimming, and Kallisto (v0.44.0) (Bray et al., 2016) to quantify gene expression levels in Transcript Per Million (TPM) normalized values. To perform clipping with Trimmomatic, sliding window size was set to 4 and mismatches was set to 2. For trimming, we set simple clip threshold to 10 and minimum required quality to 15. Reads shorter than 32 bp after quality control were discarded. For Kallisto, an index of durum wheat cv. Svevo transcripts (Maccaferri et al., 2019) was created with a k-mer size of 31, mean fragment length set to 200 and standard deviation set to 20. The resulting expression dataset was used for gene expression visualization keeping only expressed genes (TPM > 5 in at least one sample) and studies with biological replicates (≥ 3). Differential expression analyses were performed for these experiment by means of the R package DESeq2 (Love et al., 2014) (using counts instead of TPM). Genes were considered differentially expressed if up- or downregulated > 2-fold with an FDR adjusted *P* < 0.05. For the network inference, all treated samples were used, expressed genes were kept, and the resulting stress-focused dataset was given as input to GENIE3 to build a gene regulatory network (GRN) (Irrthum et al., 2010). To define GENIE3 input regulators, durum wheat transcription factors were predicted using the online tool PlantTFDB v5.0.^7^ Orthology information and functional annotation were obtained from PLAZA Monocots 4.5 (Van Bel et al., 2018). Functional enrichment was performed with GOfuncR (Grote, 2022) focusing on biological processes (BP) and excluding very general and too specific GO BP terms (10 ≤ number of annotated genes per category ≤ 1,000). Functional annotation was summarized with REVIGO (Supek et al., 2011). To investigate plant phenotypes of GRN targets, the RARGE II (Akiyama et al., 2014) database was screened to explore, for predicted targets, if mutant lines exist and if they show a significant change in registered plant traits compared to the controls.

## Results

### Identification of R2R3-MYB Transcription Factors in Durum Wheat

To identify R2R3-MYBs in the durum wheat genome, two convergent approaches were followed: BLASTP searches against the entire protein database of durum wheat cv. Svevo and HMMER analyses both on the BLASTP output and on the durum wheat proteins annotated as putative MYB TFs in the genome. The BLAST analyses used *O. sativa subsp. japonica* or *A. thaliana* MYB amino acid sequences as queries, providing a total of 507 sequences. The following HMMER analyses against the previous BLAST results and all annotated MYB proteins from the durum wheat genome, retrieved a total of 976 putative MYB sequences.

Further filtering processes were carried out to refine R2R3-MYB search. The redundant sequences of candidate genes were eliminated and the longest variant of each gene was retained, getting to 557 sequences. The presence of the characterized MYB DBDs was confirmed, and proteins carrying MYB-related or truncated MYB domains were excluded from subsequent analyses. For the longest isoforms with incomplete MYB domain, the second longest variants were retrieved and analyzed with CDD. Manual inspection was carried out to refine putative R2R3-MYB TF list. In total, 415 putative MYBs were identified in durum wheat, including 233 R2R3-MYBs, 162 MYB-related, and other classes (3R-MYBs, atypical MYB4R1, and CDC5).

Further analyses were focused on the 233 identified R2R3-MYB TFs, which were named from TdMYB1A001 to TdMYBU233 in accordance to their location and order in chromosomes (genome order: A to B, U: unannotated; chromosome order: 1–7; Supplementary Table 2).

### Characterization of R2R3 Domain

**FIGURE 1:**
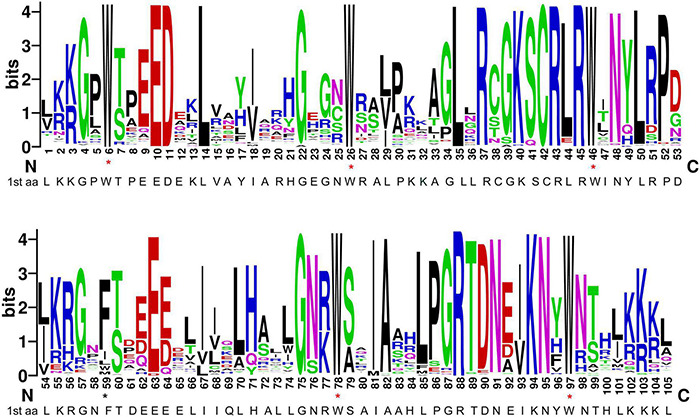
Consensus sequence and level of conservation of R2R3-MYB domains from durum wheat. The sequence logos of the R2 and R3 MYB repeats are based on full-length alignments of all domains. The height of each stack designates sequence conservation at that position and the bit score indicates the relative frequency of the corresponding acid. Conserved tryptophan residues (W) in the MYB domain are marked with red asterisks. Black asterisk highlights the position of the first canonical W in the R3 repeat replaced by hydrophobic phenylalanine or isoleucine.

R2R3 domains were aligned (Supplementary Figure 1) and the specific R2R3 sequence logo was generated (Figure 1), in order to investigate the domain structure and its level of conservation in durum wheat. The results showed that almost all homologs include five highly conserved tryptophan residues (Trp, W) in the two repeats, which play important roles in the interaction between the MYB protein and target DNA sequences (Ogata et al., 1992; Zargarian et al., 1999) and are considered as landmarks of the MYB domain. In particular, W residues are located at positions 6, 26 and 46 of the R2 repeat, and at positions 78 and 97 of the R3 repeat (Figure 1). The first and the second W residues of the R2 region are completely conserved in all durum wheat sequences. In the R3 repeat, the first W amino acid, typical of the vertebrate homologs c-MYBs, is generally replaced by hydrophobic phenylalanine (Phe, F) or isoleucine (Ile, I), as already found in many plant species (Dubos et al., 2010; Zhang et al., 2012a; Singh et al., 2020). The second and the third W residues of the R3 repeat are highly conserved, with the exception of three sequences, carrying a hydrophobic phenylalanine (TdMYB2A022 and TdMYB2B138) or tyrosine (Tyr, Y; TdMYB1B123) substitution, respectively (Supplementary Figure 1). In general, the durum wheat R2R3-MYB domain has 105 basic residues with rare deletions or insertions (Supplementary Figure 1), which were excluded from the sequence logo representation. In addition to the five highly conserved tryptophans, the residues Glu-10, Asp-11, Leu-14, Gly-22, Cys-42, Arg-43, Arg- 45 in the R2 repeat, and Glu-63, Gly-75, Ala-82, Arg-88, Lys-94, and Asn-95 in the R3 repeat are also highly conserved (Figure 1). Moreover, in durum wheat, as in other plant species, the major conserved residues in the MYB domains are mainly distributed between the second and the third conserved Trp in both repeats (Figure 1). The insertion of the leucine residue Leu-35 between the second and the third helix, can be found in 194 durum wheat proteins. Furthermore, according to previous findings, the linker region between R2 and R3 repeats carries the LRPD highly conserved motif (Williams and Grotewold, 1997; Stracke et al., 2001; Zhang et al., 2012a; Li et al., 2016, 2020c; Singh et al., 2020).

### Chromosomal Location, Gene Structure, Protein Properties, and Cellular Localization

The chromosomal location and gene structure of the identified durum wheat R2R3-MYBs were investigated. A total of 122 coding sequences (CDS) are oriented on forward strand and 111 on reverse strand. Sequence length varied from 702 bp (*TdMYB4B182* and *TdMYB4B187* genes) to 101,175 bp (*TdMYB2A030* gene). Chromosomal location analysis revealed that 119 and 108 *MYB* genes are located on the A and B genome, respectively; for six genes, location could not be determined (Supplementary Figure 2). The number of genes per chromosome ranges from 13, on chromosomes 5A and 6B, to 24 on chromosome 2A, which contains the highest number of *MYB* genes, followed by chromosome 2B, with 21 genes. The analysis of exon-intron organization of each gene revealed that most durum wheat *R2R3-MYB* genes contain few introns (0 to 3), even though, in some cases we found 4 (3 genes), 6 (2 genes) or 11 (2 genes) introns (Supplementary Table 2).

The predicted isoelectric point (pI) and molecular weight (Mw) of durum wheat R2R3-MYB proteins are reported in Supplementary Table 2. The pI ranges from 4.57 (TdMYB6B211) to 10.31 (TdMYB2B135) and the Mw varies from 22,387.62 (TdMYB2B135) to 92,544.91 Dalton (TdMYB2B138). Most of the proteins encoded by our *TdMYB* genes were predicted to have a nuclear localization. However, four of them (TdMYB1A007, TdMYB1A011, TdMYB3A058, and TdMYB1B130) were predicted to be located in the chloroplast and other four (TdMYB1A013, TdMYB5A082, TdMYB2B134, and TdMYB6B210) in the mitochondrion (Supplementary Table 2) as already observed with the same tool Cello in sunflower (Li et al., 2020b) and in chili pepper (Arce-Rodríguez et al., 2021).

### Phylogenetic and Motif Analyses

Phylogenetic studies were performed to define durum wheat MYB subfamilies, facilitate gene function prediction, and guide subsequent expression analyses. TdMYBs were processed in combination with Arabidopsis or rice R2R3-MYB TFs annotated as in Jiang and Rao (2020) and in accordance to previous functional Arabidopsis subgroups, designed from S1 to S25 (Stracke et al., 2001; Dubos et al., 2010; Katiyar et al., 2012). We took advantage of the comprehensive studies carried out by Jiang and Rao (2020), where the evolution of plant R2R3-MYB proteins was extensively explored on a broad plant and algae collection. The authors provided a new classification scheme for plant R2R3-MYBs grouped in 10 subfamilies, and compared this pattern with classification systems proposed in previous studies.

**FIGURE 2:**
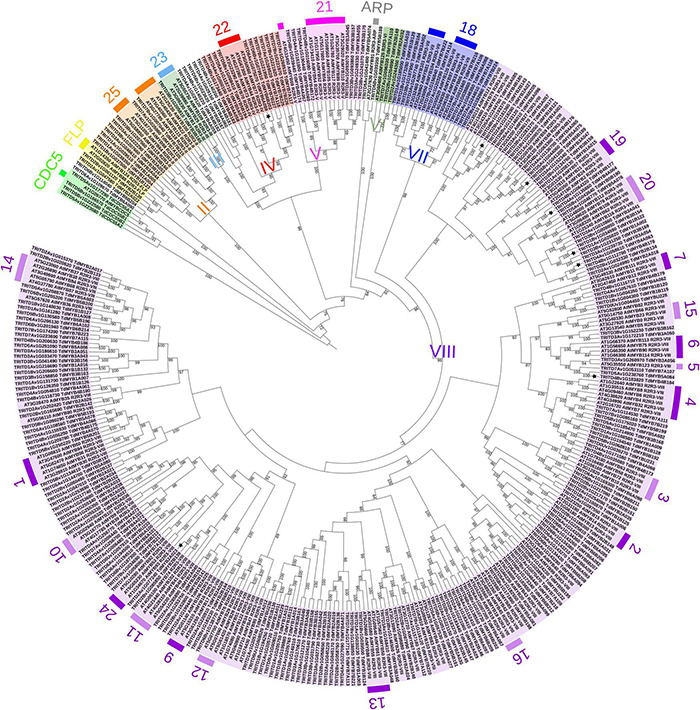
Phylogenetic analysis of R2R3-MYB proteins from durum wheat and *Arabidopsis thaliana*. Maximum Likelihood tree of 237 durum wheat (TRITD-TdMYBs) and 127 Arabidopsis (AtMYBs) R2R3-MYB TFs. The tree is rooted with CDC5 sequences. Roman numbers indicate phylogenetic groups according to Jiang and Rao, 2020; external, colored numbers refer to Arabidopsis functional clusters according to Stracke et al. (2001) and Dubos et al. (2010). Stars are referred to the MYBs analyzed by means of qPCR. Numbers on the branch nodes indicate bootstrap values (only bootstraps > 60 are shown).

**FIGURE 3:**
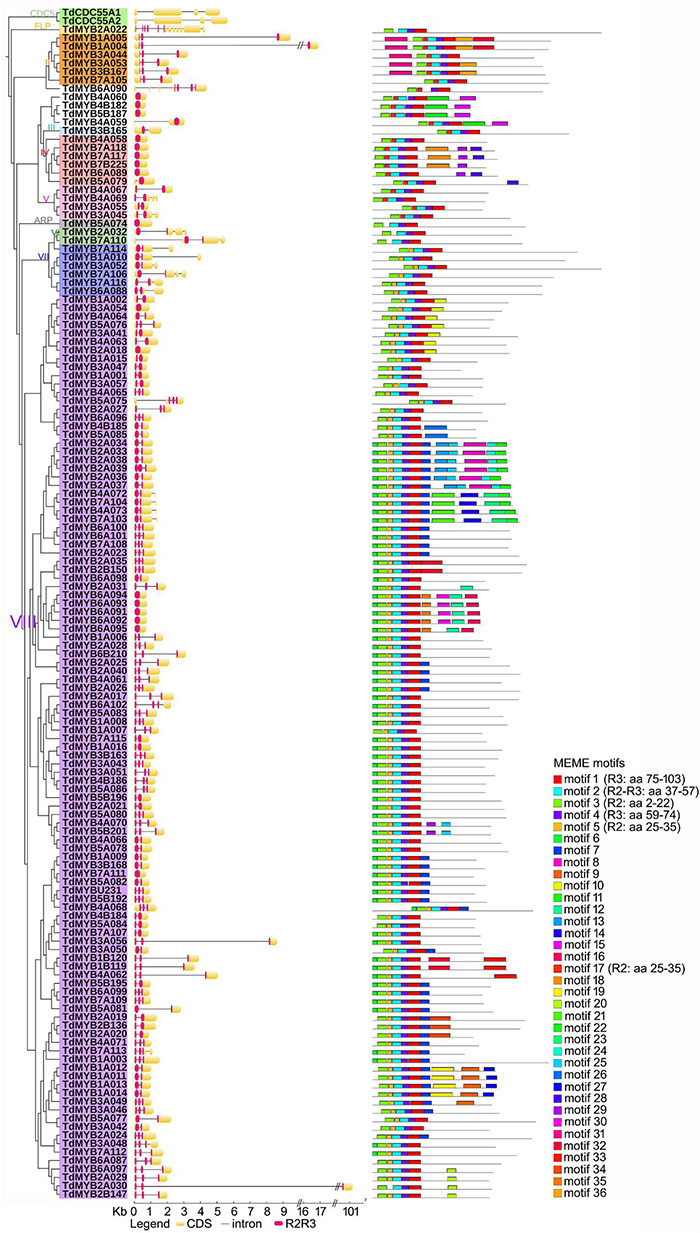
Phylogenetic relationships, gene structure, and motif composition of R2R3-MYBs in durum wheat. To facilitate reading, only one homolog for each couple was considered (141 sequences). In the phylogenetic tree (left), Roman numbers and colors indicate groups according to Jiang and Rao (2020); outgroup: CDC5 genes. In the gene structure (middle), yellow boxes, black lines and red portions designate exons, introns and R2R3 domains, respectively.

The durum wheat phylogenetic tree with Arabidopsis homologs (Figure 2^8^) was rooted with AtCDC5 and putative durum wheat CDC5 proteins, as they diverge from R2R3-MYBs (Du et al., 2015). The phylogenetic analysis placed the 233 durum wheat R2R3-MYBs into nine of the earlier identified groups (Jiang and Rao, 2020), from II to VIII, plus the FLP and ARP homologs. The clustering based on Arabidopsis was confirmed by phylogenetic analysis combining rice and durum wheat homologs (Supplementary Figure 3 and see text footnote 8). In general, durum wheat proteins fell into the 25 Arabidopsis functional groups. Interestingly, TdMYB6A090 and TdMYB6B204 occupied a more external position in the phylogenetic tree with Arabidopsis or with rice (Figure 3 and Supplementary Figure 3). Moreover, the *T. aestivum* orthologs were defined (Supplementary Figure 4).

**FIGURE 4:**
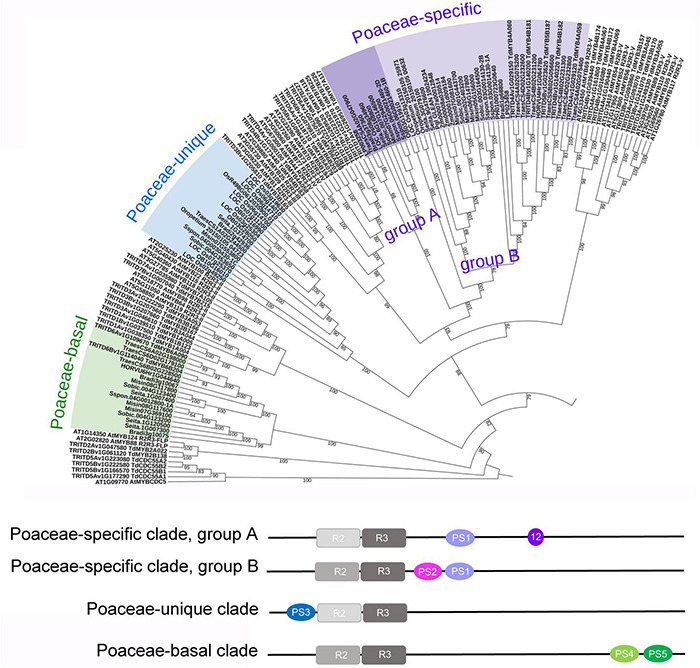
Phylogenetic relationships of Poaceae peculiar sequences with other R2R3-MYBs, and their motifs. Upper panel: a portion of the phylogenetic tree including *Arabidopsis thaliana* (127 AtMYBs), durum wheat (237 TRITD-TdMYBs), and R2R3-MYBs exclusive of the Poaceae family (77 sequences), clustering in three clades, namely Poaceae-specific (groups A and B), Poaceae-unique, and Poaceae-basal clades. Lower panel: graphic representation of the position of R2R3 and Poaceae peculiar motifs (PS1 to PS5); in Poaceae-specific clade group A, 12 refers to motif 12-Jiang. Species name abbreviations: Bradi: *Brachypodium distachyon*; Pgl_GLEAN: *Cenchrus americanus*; HORVU: *Hordeum vulgare*; LP: *Lolium perenne*; Misin: *Miscanthus sinensis*; Oropetium: *Oropetium thomaeum*; OB: *Oryza brachyantha*; LOC_Os: *Oryza sativa* subsp. *japonica*; Os: *Oryza sativa* subsp. *indica*; PH: *Phyllostachys edulis*; Sspon: *Saccharum spontaneum*; Seita: *Setaria italica*; Sobic; *Sorghum bicolor*; Traes: *Triticum aestivum*; Zm: *Zea mays*.

The topology of the rice phylogenetic tree (Supplementary Figure 3) allowed the identification of Poaceae-specific R2R3-MYBs, as described by Jiang and Rao (2020), who reported the existence of two clades found in the Poaceae, not clustering with any other known R2R3-MYB subfamily. The first clade, including two rice sequences, was named “Poaceae-specific,” and was considered divergent from subfamily IV, since some of its sequences share the motif 12 (motif 12-Jiang) with this subfamily (Jiang and Rao, 2020). The peculiarity of this clade is due to the presence of a specific motif (PS1, Supplementary Table 3, Figure 4, and see text footnote 8), which is absent in other sequences. To support this finding, we expanded the search, by seeking PS1 in all plant genomes available in PLAZA, and identified a total of 43 genes harboring a complete R2R3 domain (Supplementary Table 3). All the plant species carrying these genes belong to the Poaceae family and all the 43 genes contain the Poaceae-specific PS1 motif, while only 10 of them also include the motif 12-Jiang. In the 33 sequences without the motif 12-Jiang we identified an additional motif (PS2), correlated to (Pearson correlation coefficient = 0.6) the PS1 motif (Supplementary Table 3 and Figure 4). In order to gain a better picture about the relationships of the Poaceae-specific genes with the other R2R3-MYB genes, we added the 43 sequences to the phylogenetic tree of durum wheat with Arabidopsis or with rice, separately (Figure 4, Supplementary Figure 5, and see text footnote 8). The Poaceae-specific genes confirm to form a divergent clade stemming from R2R3-MYB group IV. In addition, this specific cluster is further divided in two groups, the first one (group A) including sequences carrying the motif 12-Jiang (except for LOC_Os03g13310, which is basal to the other group) but not the PS2 motif, and the second group (group B) including sequences with the PS2, but not the motif 12-Jiang (Supplementary Table 3 and Figure 4).

The second clade reported by Jiang and Rao (2020) was named “Unique Poaceae R2R3-MYB” and included six rice sequences. To support this result, we extended the analysis and identified 19 genes where we detected the PS3 motif, except for LOC_Os02g49250 (Supplementary Table 3 and Figure 4). All the plant species carrying these genes belong to the Poaceae family, with only one representative of the Triticeae, i.e., one sequence from *T*. *aestivum*, while no durum wheat sequences are present in this group. In the phylogenetic tree, this set of Poaceae-unique sequences is clearly separated from R2R3-MYB group III, IV, and V (Figure 4, Supplementary Figure 5, and see text footnote 8).

Besides the specific and unique sets of sequences described above, we observed that two durum wheat R2R3-MYBs (TdMYB6B204 and TdMYB6A090) occupied a more basal position in the phylogenetic trees, being placed between CDC5 and FLP (Arabidopsis tree), or between FLP genes and R2R3-MYB group II (rice tree) (Figure 3 and Supplementary Figure 3). In these sequences, we identified a peculiar R2R3-MYB domain. When searching in PLAZA, we retrieved a total of 17 sequences, all belonging to Poaceae species, most of which (15) carrying two distinctive, newly identified motifs (PS4 and PS5, Supplementary Table 3). All these 17 sequences were added to the phylogenetic trees and occupied the same position, between FLP and group II (Figure 4, Supplementary Figure 5, and see text footnote 8).

Phylogeny information allowed the attribution of putative functions to durum wheat R2R3-MYB TFs based on functional clusters in Arabidopsis (Matus et al., 2008; Dubos et al., 2010). The largest subfamily of R2R3-MYBs in land plants is represented by group VIII (Jiang and Rao, 2020), which includes more than half of the durum wheat sequences, namely 179 proteins (Figure 3). TdMYBs belonging to group VIII cluster with 18 Arabidopsis subfamilies, whose members are involved in several processes (Dubos et al., 2010). Group II includes nine TdMYBs (S25 family), while group III contains only one durum wheat homolog, similar to S23 Arabidopsis members. Group IV, V, and VII comprise durum wheat MYB members clustering with Arabidopsis S22, S21, and S18 subfamilies, respectively. Three TdMYBs cluster with AtMYB125/DUO1 (group VI), while two durum sequences group with Arabidopsis ARPs. Finally, two putative durum FLP R2R3-MYBs are grouped with their Arabidopsis homologs.

We used the online program MEME to search for conserved motifs shared by TdMYB TFs to further support and define durum wheat R2R3-MYB subfamilies (Supplementary Table 4). For ease of visualization, we focused our analysis on one for each homolog couple (139 TdMYBs in total, Figure 2), starting from genome A-localized MYBs and considering the corresponding B or U homologs when related TdAMYB was missing. We processed the entire sequences, to further highlight similarities also in the R2R3 repeats, among members of the same group. A total of 36 motifs were searched (Supplementary Table 4 and Figure 2). Six of these motifs (from 1 to 5, and motif 17) represent portions of conserved regions within the R2R3-MYB domains. Motifs 3, plus 5 or 17, and left part of motif 2 compose the R2 repeat, while right part of motif 2, motif 4 and 1 form the R3 repeat (Figure 2). Motifs 1–4 are conserved in almost all TdMYBs; motif 17 is conserved in the MYB domain of proteins belonging to groups II to V, while group VII and VIII members contain motif 5 in place of motif 17 (Figure 2). ARP and group VI members possess neither motif 5 nor 17. In addition to the highly conserved motifs of MYB R2 and R3 repeats, TdMYB members within the same clade share other auxiliary motifs. For instance, motifs 6 and 7 are present and highly conserved only in group VIII, and correspond to the previously identified motifs 31 and 32, respectively (Jiang and Rao, 2020), motif 7 being shared by subfamily S24 (Dubos et al., 2010). Motif 11 (subfamily S10) characterizes a clade of 10 sequences in group VIII (Figure 2). Motif 18 and motif 28 (motif 10 and 14 in Jiang and Rao, 2020, respectively) are only found in group IV.

### Gene Expression Profile in Durum Wheat Tissues Under Abiotic Stress Conditions

**FIGURE 5:**
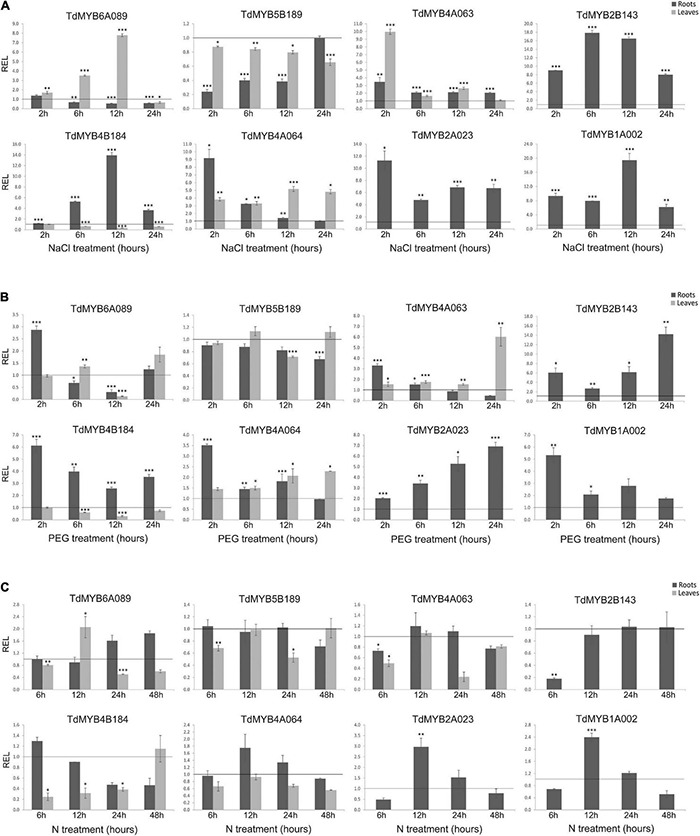
Relative expression levels (real-time quantitative PCR) of eight R2R3-MYB genes in durum wheat roots and leaves. Plants were subjected to salt **(A)**, drought **(B)**, or nitrogen (N) deprivation **(C)** stress, in time course experiments. Relative expression was calculated at each time point for stressed sample with respect to the corresponding control (non-stressed sample). The control expression value at each time point is set as 1 and is represented by the black line in each graph. *, **, and *** indicate significance at *p* < 0.1, 0.05, and 0.01, respectively. REL, relative expression level.

Eight *R2R3-MYB* genes (*TdMYB1A002*, *TdMYB2A023*, *TdMYB4A063*, *TdMYB4A064*, *TdMYB6A089*, *TdMYB2B143*, *TdMYB4B184*, and *TdMYB5B189*) were selected since they fall in the abiotic stress response clusters of Arabidopsis (Figure 3), or their homologs have been previously characterized. Their relative expression levels were analyzed in durum wheat plants subjected to salt or drought stress treatments and nitrogen deprivation in time-course experiments, since, on the bases of previous results in other species (see Introduction section), we would expect a possible involvement of these *TdMYB* genes in response to the above stresses. Figure 5 reports the results of qPCR expression analysis for the eight genes, where the control expression value at each time point is represented by the black line set to 1 in each graph.

In Figure 5A results for salt stress are shown. The *TdMYB6A089* transcript abundance gradually increased in the leaves from 6 h (≅3.5-fold) to 12 h of treatment (≅7.8-fold). This transcript showed no relevant expression changes in roots. The *TdMYB5B189* transcript was significantly downregulated in roots from 2 h (≅4.0-fold) to 12 h of treatment (≅2.6-fold), although it showed no evident expression changes in leaves. The *TdMYB4A063* transcript was always upregulated in roots during the treatment with a maximum of ≅3.4-fold at 2 h. In leaves as well, the transcript showed an immediate and marked increase of expression at 2 h of treatment (≅10-fold). An increased abundance of this transcript was also detected at 12 h (≅2.6-fold). The *TdMYB4B184* transcript showed an opposite trend in the two analyzed tissues. In stressed roots, it started from control expression levels, then increased linearly until reaching the maximum level at 12 h (≅14.0-fold), then decreased at 24 h, remaining upregulated (≅3.6-fold). In leaves instead, it started from control expression levels at 2 h, then decreased at 6 and 12 h, showing ≅3.2-fold downregulation. The *TdMYB4A064* transcript showed a rapid and significant increase of expression in roots at 2 h of treatment (≅9.0-fold), then it decreased at 6 h (≅3.3-fold) and returned to control levels at 12 and 24 h. This transcript was also upregulated in leaves during the salt treatment, showing a maximum fold-change value at 12 and 24 h (≅5.0- and 4.8-fold, respectively). Three of the eight analyzed gene transcripts, *TdMYB2B143*, *TdMYB2A023*, and *TdMYB1A002* were significantly and abundantly upregulated in stressed roots at all time-points. Although we noticed gene activation for *TdMYB2A023* and *TdMYB1A002* in stressed leaves, we could not quantify the relative expression, since expression levels were untraceable (low expressed/no expressed) in control leaves. *TdMYB2B143* showed no expression in both control and stressed leaves.

Following drought stress (Figure 5B), the *TdMYB6A089* transcript expression in leaves was significantly downregulated only at 12 h, while in roots it was significantly upregulated (≅2.9-fold) at 2 h of treatment and downregulated at 12 h (≅3.3-fold). The *TdMYB5B189* transcript showed no change in either analyzed tissues. The expression of *TdMYB4A063* in roots was significantly higher than the control at 2 h (≅3.3-fold), then gradually decreased until reaching a downregulation (≅2.2-fold) at 24 h, whereas in leaves the expression levels increased significantly at 24 h (≅6.0-fold). *TdMYB4B184* transcript was significantly upregulated in roots with a maximum at 2 h (≅6.0-fold), but its expression was significantly downregulated in leaves at 12 h (≅3.3-fold). The analysis of the *TdMYB4A064* transcript in stressed roots displayed a significant increase in expression levels (≅3.5-fold) at 2 h. In leaf tissues, the expression levels increased at 12 and 24 h (≅2.0-fold in both time points). Even in this treatment, *TdMYB2B143*, *TdMYB2A023*, and *TdMYB1A002* transcripts, almost undetectable in leaf tissues, showed high expression levels in stressed roots. In particular, TdMYB2B143 and *TdMYB2A023* were significantly upregulated at all time points, while TdMYB1A002 showed a significantly higher expression at 2 h (≅5.3 fold) and then its expression decreased.

The effects of N deprivation on the expression levels of the tested genes were much less marked compared to the other stresses (Figure 5C). *TdMYB6A089* transcript showed no expression change in this condition, except for a slight but significant increase at 12 h in leaves (≅2.0-fold). The *TdMYB5B189* transcript levels were similar to controls in both tissues at all time points, without relevant changing. The *TdMYB4A063* transcript showed no expression change in roots, while in leaves it was downregulated at 6 and 24 h of treatment, with a more evident decrease at 24 h (≅4.0-fold). The *TdMYB4B184* transcript was significantly downregulated in leaves from 6 h (≅4.0-fold) to 24 h (≅2.6-fold). A weak downregulation was also detected in treated roots at 24 and 48 h (≅2.0-fold at both time-points). The expression analysis of the *TdMYB4A064* transcript showed no significant changes in this stress treatment. The three genes, *TdMYB2B143*, *TdMYB2A023*, and *TdMYB1A002* were barely detectable in control and stressed leaves. In roots, *TdMYB2B143* expression was significantly lower at 6 h (≅6.0-fold) and then the expression levels reverted to control values. The *TdMYB2A023* expression levels showed a ≅2.0-fold decrease at 6 h, a significant ≅3.0-fold increase at 12 h, and control-like expression values at 24 and 48 h. *TdMYB1A002* transcripts showed a ≅2.0-fold expression levels decrease at 6 h, a significantly increase at 12 h (≅2.4-fold) and then the expression returned to the control levels (Figure 5C).

### A Regulatory View on the Selected TdR2R3-MYBs

We screened scientific literature to retrieve transcriptomic studies focused on durum wheat response to abiotic stresses. We selected six studies (Supplementary Table 5) addressing durum wheat response to several stresses including drought, heat, a combination of heat and drought, and nitrogen deprivation in different tissues, namely roots, leaves, spikes and grains, amounting to 63 RNA-seq samples (28 control and 35 treated samples). Raw data were processed and filtered keeping only expressed genes and obtaining 40,333 genes, 141 of these were TdR2R3-MYBs identified in this study. The remaining 92 TdR2R3-MYBs were not retained because probably not expressed in the tissues and/or the conditions included in the expression dataset.

**FIGURE 6:**
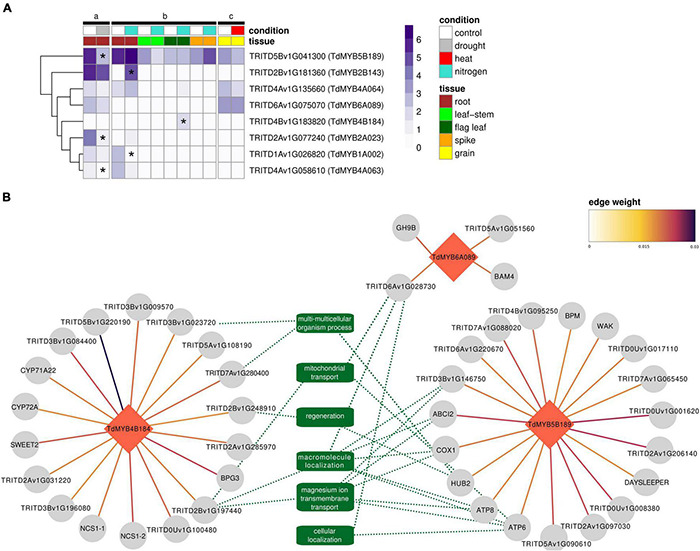
Gene expression and regulatory interactions in public transcriptomic data on abiotic stresses. **(A)** Expression profiles of the eight TdR2R3-MYB genes selected for qPCR experiments across a subset of the public transcriptomic studies (a-c) listed in Supplementary Table 5 for which ≥3 replicates were available. Expression values are given as log_2_(TPM + 1) of the average of the biological replicates per condition per study. Differentially expressed genes are marked with an asterisk. **(B)** GENIE3 regulatory network of *TdMYB5B189*, *TdMYB4B184*, and *TdMYB6A089* TFs (red diamonds). Target genes are depicted with gray circles. Links between genes show edge weight. Green dotted lines show connections with related REVIGO-summarized biological processes in common across regulatory clusters (minimum two). Where available, gene name (from *Arabidopsis thaliana* ortholog) in place of gene ID is shown.

All eight genes selected for our qPCR experiments passed the filter to be included in the expression dataset, so their absolute expression levels (as TPM values) in response to abiotic stresses could be visualized (Figure 6A). Differential expression analyses showed significant downregulations for *TdMYB5B189*, *TdMYB2A023*, and *TdMYB4A063* in root tissues in response to drought stress. Under nitrogen stress, upregulation was observed for *TdMYB2B143* in roots and *TdMYB4B184* in flag leaves, while downregulation was observed for *TdMYB1A002* in roots. No significant changes were observed in spike or grain tissues for the selected genes in the analyzed stresses. A list of regulators for durum wheat to supply to GENIE3 was predicted with PlantTFDB. This tool led to the identification of a total of 3,532 TFs. After filtering for TFs present in a stress-focused expression dataset, 1,812 TFs were retained. These TFs were used as input regulators for a GENIE3 GRN construction to extract the sub-networks of our eight genes selected for qPCR experiments. From the GRN analyses, only top interactions were kept (16,000 edges ranked by weight). This list included three (*TdMYB4B184*, *TdMYB5B189*, *TdMYB6A089*) out of the eight selected durum wheat R2R3-MYBs, with 41 interactions, representing putative targets (Figure 6B and Supplementary Table 6).

*TdMYB4B184* showed interactions with 18 genes (Figure 6B). This cluster did not show any significant GO enrichment. Nevertheless, among these 18 genes, interestingly, five had orthologs in Arabidopsis. Overexpression lines for Arabidopsis orthologs showed a phenotype linked to plant size (rosette leaf, stem, and whole plant measures), as in the cases of *BPG3* (ortholog to TRITD3Av1G213390), *SWEET2* (ortholog to TRITD3Av1G171110), and *AT2G40400* (ortholog to TRITD3Bv1G196080), and seed number, as in the case of *NCS1* (ortholog to TRITD7Av1G160600 and TRITD7Bv1G118700). Several of these genes are in fact linked with chloroplast processes (*NCS1*, *BPG3*). Other predicted targets were protein kinases, for example TRITD5Bv1G220190, which had the strongest weight among all predicted targets of the TdR2R3-MYBs tested in this study (Figure 6B). One member of GDSL esterase/lipase G family (TRITD3Bv1G084400) was also predicted. This family has been linked with salinity and drought tolerance (Su et al., 2020). *TdMYB5B189* showed interactions with 19 genes (Figure 6B). This cluster showed a significant enrichment (FWER < 0.05) for several GO biological processes all descendants (child terms) of ion transport (GO:0006810) and for ATP metabolic process (GO:0046034). Among them, two genes encoding for ATPase subunits (*ATP6*, *ATP8*), one for cytochrome c biogenesis (*ABCI2*), and one E3 ubiquitin-protein ligase BRE1-like 2 (*HUB2*) were found. Other predicted targets were TRITD3Bv1G006290, ortholog to Arabidopsis *BPM*, known to be involved in response to salt stress, and TRITD6Av1G005530, ortholog to Arabidopsis wall associated kinases (*WAKs*), hypothesized to be salt-stress sensors participating to salt stress acclimation (Le Gall et al., 2015). *TdMYB6A089* showed interactions with only four genes (Figure 6B). Among them a beta-amylase was found, which is known to be involved in heat and cold stresses (Kaplan and Guy, 2004).

## Discussion

The MYB gene family is one of the largest groups of TFs in plants, widely explored in several plant species, including bread wheat (Wei et al., 2020; Xiao et al., 2020). In this study, a genome-wide analysis on R2R3-MYB TFs was performed in durum wheat, highlighting sequence features, phylogenetic relationships and response to abiotic stresses.

The major conserved residues in the durum wheat MYB domain, mainly distributed between the second and the third conserved Trp residues in both repeats, reflect what was previously observed in plants (Jiang and Rao, 2020). The insertion of the leucine residue (L35) in the R2 repeat, the hydrophobic residue at the beginning of R3 domain, and the R2R3 linker region are considered important steps for the origin and the evolution of plant-specific R2R3-MYB proteins (Williams and Grotewold, 1997; Stracke et al., 2001; Zhang et al., 2012a; Li et al., 2016, 2020c; Wei et al., 2020).

On the other hand, the presence of some amino acid insertions/substitutions at specific sites of the R2 and R3 repeats, may characterize subfamily and/or lineage-specific features (Du et al., 2015).

The identification of nine subfamilies of R2R3-MYBs in durum wheat, as observed in a wide range of land plants, confirms that groups of genes in this family have undergone expansion and functionalization events (Jiang and Rao, 2020). Indeed, durum wheat R2R3-MYBs falling in Arabidopsis functional groups are expected to share similar biological roles. For instance, TdMYBs belonging to group VIII are possibly involved in: response to environmental stress (S1, S2, S11, S20), regulation of phenylpropanoid biosynthesis (S4, S5, S6, and S7), cell fate and identity (S15 and S9), regulation of plant development (S14, S16, S18, S19), control of cell wall biosynthesis (S3 and S13), and plant defense (S12) (Dubos et al., 2010). The S25 family in group II may be involved in embryogenesis, while the S23 members in group III, as their Arabidopsis orthologs, might regulate salt stress and be involved in abscisic acid response (Beathard et al., 2021). Group IV, V, and VII include MYB members possibly implicated in the regulation of plant development (S22, S21, and S18). Group VI includes AtMYB125/DUO1, involved in cell cycle regulation (Cominelli and Tonelli, 2009). ARP cluster contains wheat MYBs probably playing a role in cell division processes (Sun et al., 2002), while FLP R2R3-MYBs may be implied in stomatal differentiation and patterning (Dubos et al., 2010).

By searching orthogroups and performing subsequent phylogenetic analyses, we were able to substantiate the existence of Poaceae-specific and unique R2R3-MYB TFs on a wider number of Poaceae species, including durum wheat. These sequences, forming two main clusters diverging from groups III and IV, can be considered fast evolving genes (Jiang and Rao, 2020). In addition, we detected a further gene cluster exclusive of the Poaceae species, located in a more basal position in the tree, which can be ascribed to the R2R3-MYB TFs, thus representing another peculiarity for the Poaceae family. Indeed, although the main diversification of R2R3-MYB occurred before the monocot and dicot split, each lineage continued to expand, resulting in a multiplicity of lineage-specific clades, which may contribute to functional divergence (Sun et al., 2002; Du et al., 2015; Millard et al., 2019; Jiang and Rao, 2020; Li et al., 2020a; Singh et al., 2020).

MYB transcription factors have shown to respond to environmental stimuli in several plant species. *TdMYB6A089* displays a high level of similarity with bread wheat *TaMYB1* (99% identity). *TaMyb1* expression levels changed under various abiotic treatments, registering an increase in roots under drought and salt stress (Lee et al., 2007), somehow differing from what observed here for *TdMYB6A089*, considering that growth conditions and physiological stages were not the same.

*TdMYB4B184*, which is ortholog to *TaMYB82* (99% identity), showed an overall upregulation in drought-stressed roots and leaves (at least at certain time points). In common wheat, *TaMYB82* was upregulated in seedlings during PEG treatment mimicking drought (Zhao et al., 2017). Upregulation was also observed in durum wheat from our *in silico* RNAseq analyses in flag leaves under nitrogen stress (Figure 6A), which leads to hypothesize that this gene is involved in a broader range of abiotic stresses.

*TdMYB2A023*, highly activated during drought and salt stress in durum wheat (Figure 5), but downregulated in durum wheat (cultivar Kiziltan) roots subjected to long-term drought stress (Figure 6), is 98% similar to *TaMYB80* or *TaMYB74*. Bi et al. (2016) reported that *TaMYB74* was upregulated by both rapid dehydration and slowly developing drought, in accordance with the data obtained for their Arabidopsis counterparts, *AtMYB41* and *AtMYB96*.

*TdMYB1A002*, upregulated in response to drought and salt stress in our study, is highly similar (>98% identity) to *TaPIMP1* and a *MYB2-like* gene in *T. aestivum*. *TaPIMP1* is an important positive mediator in wheat defense responses to *B. sorokiniana* and drought stress (Zhang et al., 2012c). In summary, upon *B. sorokiniana* infection and drought stress, *TaPIMP1* was upregulated, activating the defense- and stress-related genes in the ABA- and SA-signaling pathways, leading to enhanced resistance to both biotic and abiotic stress. *TaPIMP1* gene expression was upregulated under low N in root tissues of HD2967 genotype (Mahmoud et al., 2020). On the other hand, our qPCR results showed an increase at 12 h of N starvation followed by a decrease in the subsequent time points. This downregulation might be in line with what observed in adult durum wheat plants after long-term N deprivation (Figure 6A).

The target genes for *TdMYB6A089*, *TdMYB4B184*, and *TdMYB5B189* identified through the gene regulatory network were mostly linked with biological processes involved in transport and phosphorylation. These processes participate to maintain the biological membrane potential and activate signaling cascades involved in abiotic stress responses (Conde et al., 2011). It has been previously observed that a substantial proportion of orthologous gene pairs have conserved coexpression across Arabidopsis and rice implying that gene modules, also involved in abiotic stress response, are often conserved across dicots and monocots (Movahedi et al., 2011; Obertello et al., 2015; Vercruysse et al., 2020). On the other hand, it is established that recent and ancestral duplications caused an expansion of R2R3-MYBs which might result in neo- and sub-functionalization events and gene regulatory network evolution, even at the species level (Agarwal et al., 2016; Gordon et al., 2017; Jiang and Rao, 2020).

One of the interesting targets of *TdMYB5B189* is a E3 ubiquitin ligases (ortholog of Arabidopsis *HUB2*), involved in monoubiquitination of histone H2B. *HUB2* has been recently shown to be involved in stress regulation, in fact its overexpression increased the expression of drought-related genes in transgenic cotton plants (Chen et al., 2019). TRITD6Av1G220670, another target of *TdMYB5B189*, is a member of Ankyrin repeat family. Members of this gene family have been shown to be involved in response to drought and salt stresses in Arabidopsis and soybean (Zhao et al., 2020a). Within the predicted targets for *TdMYB4B184*, TRITD3Bv1G009570, a leucine-rich repeat receptor-like protein kinase family protein, is ortholog to AT2G34930, which was suggested to respond to both abiotic and biotic stresses (Ma and Bohnert, 2007).

Taken together, the present study provides a first insight into the landscape of durum wheat R2R3-MYBs, which, along with the experimental analyses and the unveiled regulatory dynamics, constitutes a solid base to further explore the roles of this gene family in the response to abiotic stresses and select valuable candidates to study for future durum wheat plant breeding programs.

## Data Availability Statement

The original contributions presented in the study are included in the article/Supplementary Material, and https://github.com/integrativeplantbiology/R2R3-MYBs-in-durum-wheat, further inquiries can be directed to the corresponding authors.

## Author Contributions

EB and GS: conceptualization, methodology, and supervision. EB: investigation, data curation, and visualization. PC: investigation, methodology, and data curation. AM, AS, and DZ: formal analysis. EB, PC, and GS: writing—original draft, writing—review, and editing. All authors contributed to the article and approved the submitted version.

## Conflict of Interest

The authors declare that the research was conducted in the absence of any commercial or financial relationships that could be construed as a potential conflict of interest.

## Publisher’s Note

All claims expressed in this article are solely those of the authors and do not necessarily represent those of their affiliated organizations, or those of the publisher, the editors and the reviewers. Any product that may be evaluated in this article, or claim that may be made by its manufacturer, is not guaranteed or endorsed by the publisher.

## Footnotes

1
http://planttfdb.gao-lab.org/

2
http://pfam.xfam.org/family/PF00249

3
http://hmmer.org/download.html

4
https://wheat.pw.usda.gov/jb/?data=/ggds/whe-svevo2018

5
https://mafft.cbrc.jp/alignment/server/

6
https://primer3plus.com/

7
http://planttfdb.gao-lab.org/prediction.php

8
https://github.com/integrativeplantbiology/R2R3-MYBs-in-durum-wheat
