# Supplementary material for: R2R3-MYBs in Durum Wheat: Genome-Wide Identification, Poaceae-Specific Clusters, Expression, and Regulatory Dynamics Under Abiotic Stresses
Source: Front Plant Sci. 2022 Jun 20;13:896945. doi: 10.3389/fpls.2022.896945 (PMC9252425; doi:10.3389/fpls.2022.896945)
Supplement: Supplementary file 1 [file Table_1.DOCX]

**Supplementary** **Table 1**. Primers used for qPCR expression analysis

| Gene ID | Gene name | Forward 5’-3’ | Reverse 5’-3’ | Product length (bp) |
| --- | --- | --- | --- | --- |
| TRITD6Av1G075070.1 | TdMYB1A002 | CGCGCCAGGACGAGGAT | GCGCCGGTGGAGGAGA | 135 |
| TRITD5Bv1G041300.1 | TdMYB2A023 | GTCACCCTCATCATCTTCATC | GCAGGCGTGCCCTTCAAT | 135 |
| TRITD2Bv1G181360.1 | TdMYB2B143 | GACGTGCTGGACTTCCTGTG | GACCCATACTCCTCGCTCGT | 98 |
| TRITD4Av1G058610.1 | TdMYB4A063 | CATATGCGACACGGACCAG | AAGCTGCCCATCTCGAAGTC | 129 |
| TRITD4Bv1G183820.1 | TdMYB4A064 | GGAGGAGACGCAGATGAGTG | ACTTGACCCAGTCCCAGTCC | 127 |
| TRITD4Av1G135660.2 | TdMYB4B184 | GAGGGGACTGGATGCAGGAA | AGCCGAAACGCCGACGTC | 140 |
| TRITD2Av1G077240.1 | TdMYB5B189 | CCCACGACCGAACAGCAG | GCACCATGTGATCCTGGTGTA | 187 |
| TRITD1Av1G026820.1 | TdMYB6A089 | GATGGCTCGATGATGCAGTG | CCGAACTCAAAGTCCGGGATA | 137 |
|  | RLI(a) | TTGAGCAACTCATGGACCAG | GCTTTCCAAGGCACAAACAT | 84 |
