# Supplementary material for: R2R3-MYBs in Durum Wheat: Genome-Wide Identification, Poaceae-Specific Clusters, Expression, and Regulatory Dynamics Under Abiotic Stresses
Source: Front Plant Sci. 2022 Jun 20;13:896945. doi: 10.3389/fpls.2022.896945 (PMC9252425; doi:10.3389/fpls.2022.896945)
Supplement: Supplementary file 7 [file Image_1.PDF]

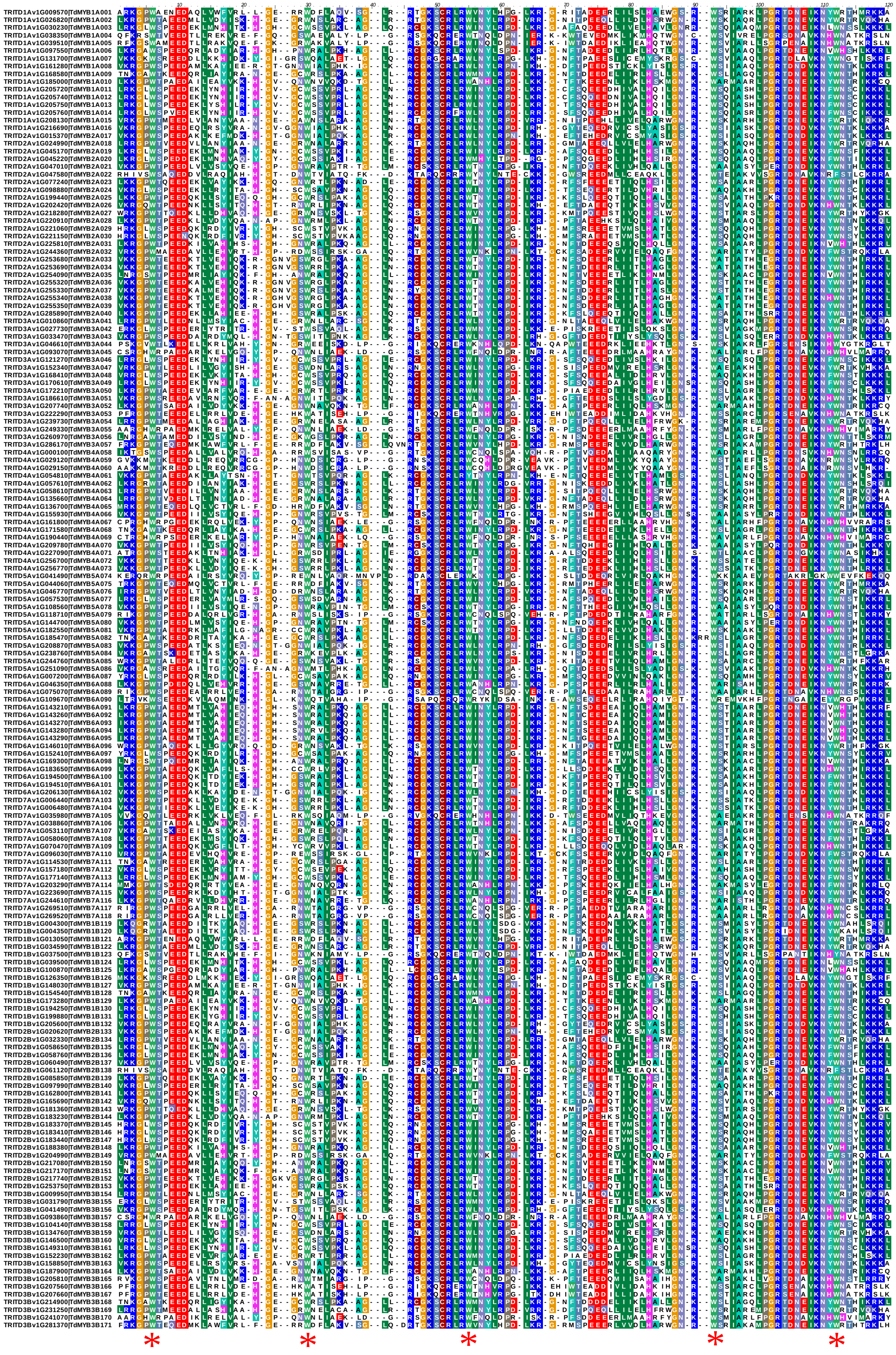

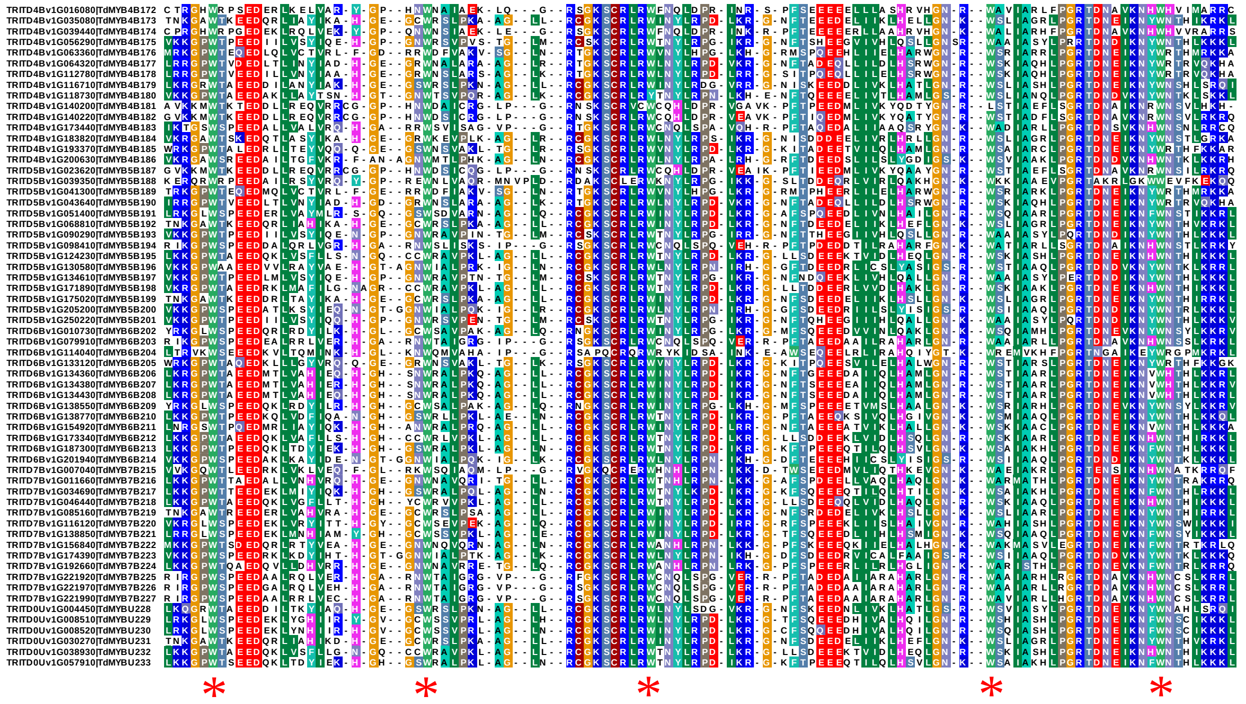

Supplementary Figure 1. Multiple sequence alignment of 233 durum wheat R2R3-MYB domains, visualized with Bioedit. Asterisks highlight the sites of the five landmark tryptophan residues (W) in the repeat regions.
