# Supplementary figures and images for: R2R3-MYBs in Durum Wheat: Genome-Wide Identification, Poaceae-Specific Clusters, Expression, and Regulatory Dynamics Under Abiotic Stresses
Source: Front Plant Sci. 2022 Jun 20;13:896945. doi: 10.3389/fpls.2022.896945 (PMC9252425; doi:10.3389/fpls.2022.896945)

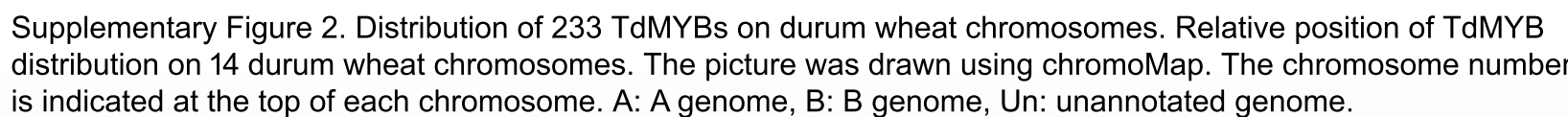

Supplement: Supplementary file 8 [file Image_2.PDF]
