# Supplementary material for: R2R3-MYBs in Durum Wheat: Genome-Wide Identification, Poaceae-Specific Clusters, Expression, and Regulatory Dynamics Under Abiotic Stresses
Source: Front Plant Sci. 2022 Jun 20;13:896945. doi: 10.3389/fpls.2022.896945 (PMC9252425; doi:10.3389/fpls.2022.896945)
Supplement: Supplementary file 10 [file Image_4.PDF]

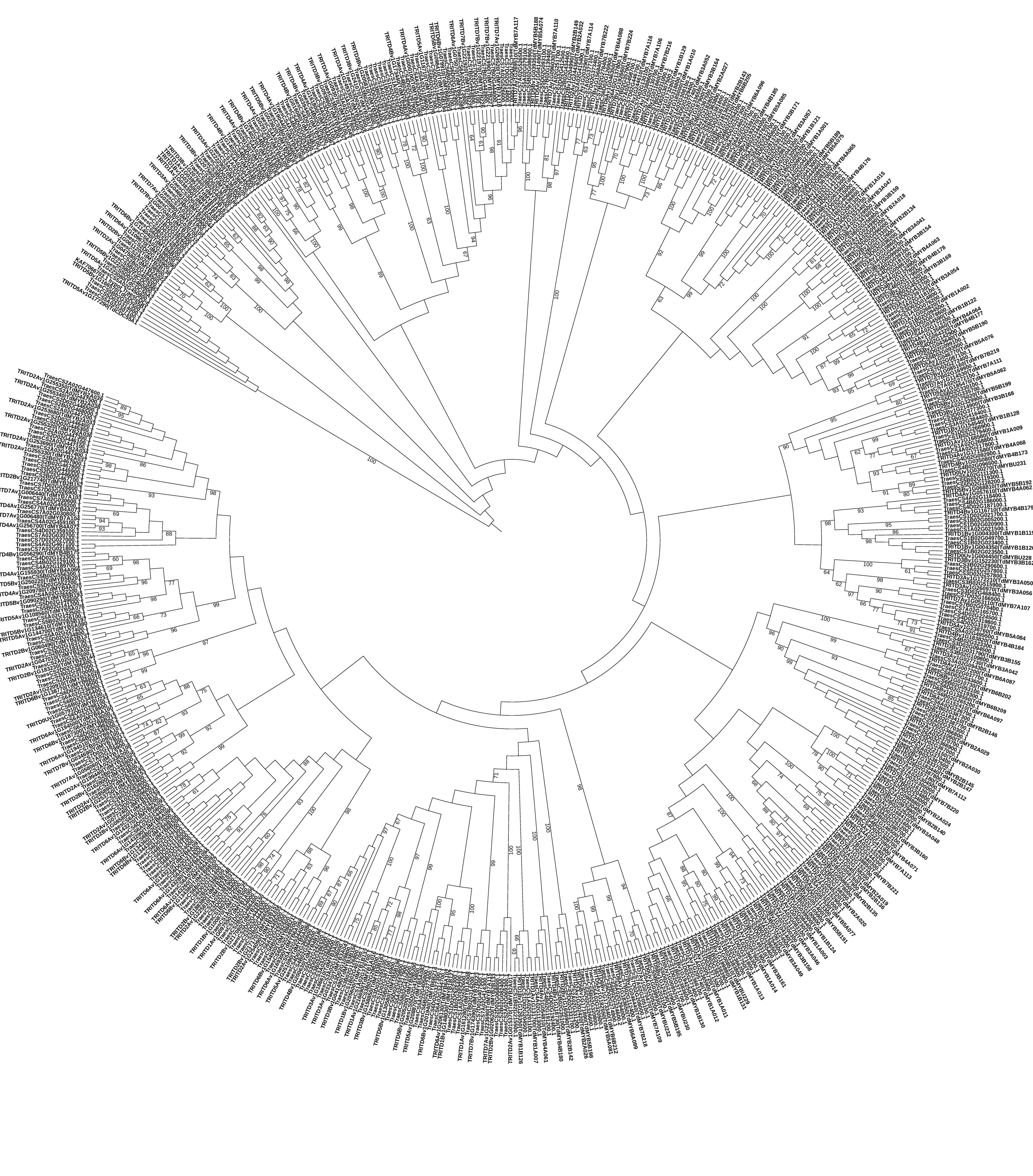

Supplementary Figure 4. Phylogenetic analysis of R2R3-MYB proteins from durum wheat and Triticum aestivum. Maximum Likelihood tree is rooted with CDC5 sequences. Numbers on the branch nodes indicate bootstrap values (only bootstraps >60 are indicated).
