# Supplementary material for: R2R3-MYBs in Durum Wheat: Genome-Wide Identification, Poaceae-Specific Clusters, Expression, and Regulatory Dynamics Under Abiotic Stresses
Source: Front Plant Sci. 2022 Jun 20;13:896945. doi: 10.3389/fpls.2022.896945 (PMC9252425; doi:10.3389/fpls.2022.896945)
Supplement: Supplementary file 11 [file Image_5.PDF]

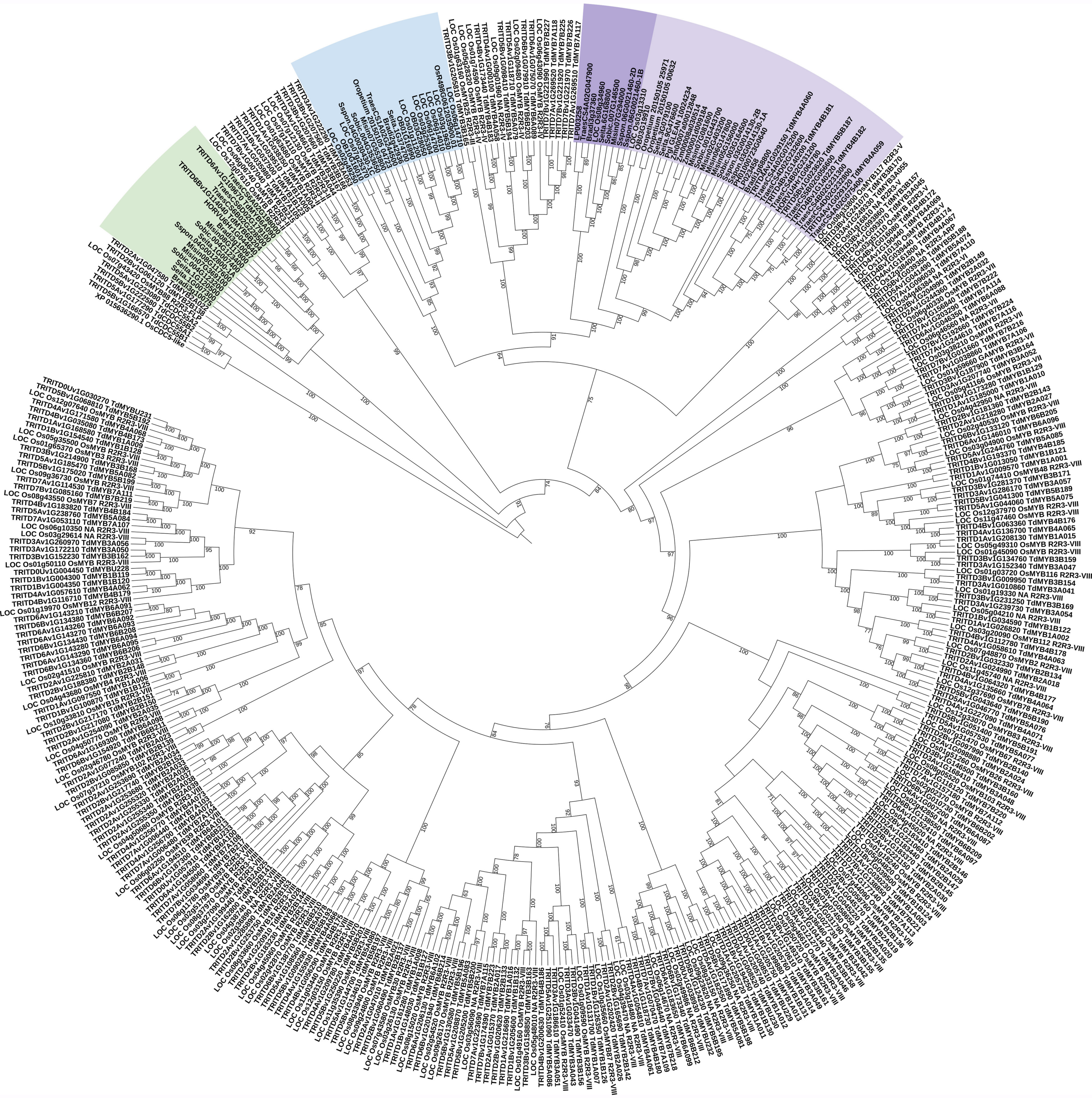

Supplementary Figure 5. Phylogenetic relationships of Poaceae peculiar sequences with other rice MYBs. Phylogenetic tree including rice, durum wheat R2R3-MYBs, and R2R3-MYBs exclusive of the Poaceae family, clustering in three clades, namely Poaceae-specific (purple), Poaceae-unique (blue), and Poaceae-basal (green) clades.
